# Supplementary material for: The Multikinase Inhibitor AD80 Induces Mitotic Catastrophe and Autophagy in Pancreatic Cancer Cells
Source: Cancers (Basel). 2023 Jul 29;15(15):3866. doi: 10.3390/cancers15153866 (PMC10417629; doi:10.3390/cancers15153866)
Supplement: Supplementary file 1 [file cancers-15-03866-s001.zip › Figure S1.pdf]

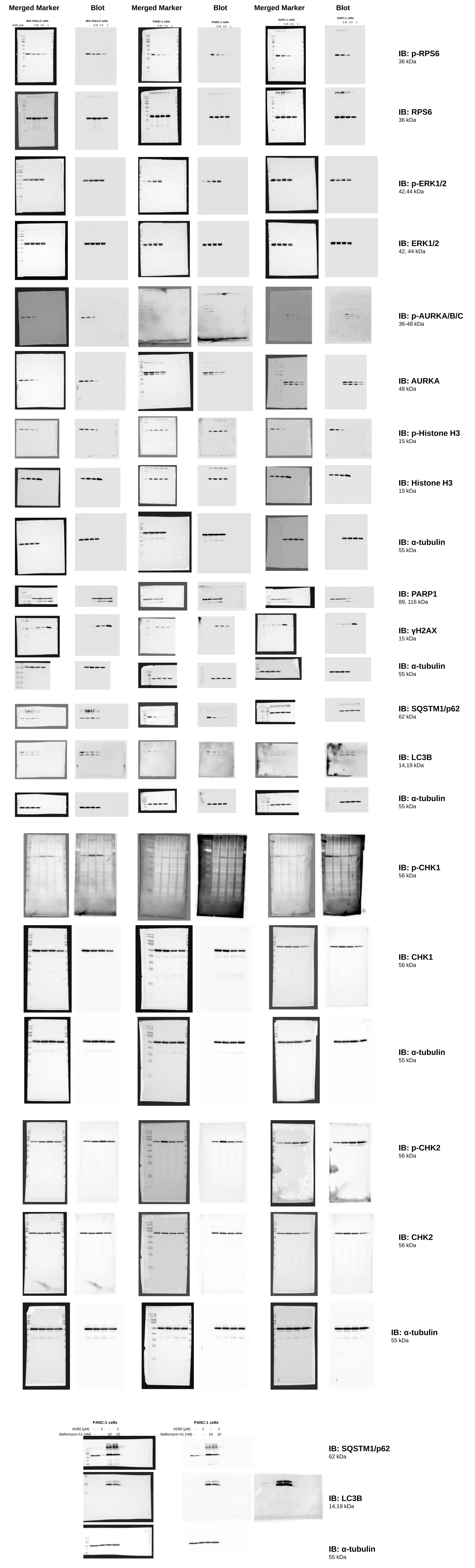
Merged Marker
Blot
Merged Marker
Blot
Merged Marker
Blot
MIA PaCa-2 cells
PANC-1 cells
AsPC-1 cells
IB: p-RPS6
36 kDa
IB: RPS6
36 kDa
IB: p-ERK1/2
42,44 kDa
IB: ERK1/2
42, 44 kDa
IB: p-AURKA/B/C
36-48 kDa
IB: AURKA
48 kDa
IB: p-Histone H3
15 kDa
IB: Histone H3
15 kDa
IB: α-tubulin
55 kDa
IB: PARP1
89, 116 kDa
IB: γH2AX
15 kDa
IB: α-tubulin
55 kDa
IB: SQSTM1/p62
62 kDa
IB: LC3B
14,19 kDa
IB: α-tubulin
55 kDa
IB: p-CHK1
56 kDa
IB: CHK1
56 kDa
IB: α-tubulin
55 kDa
IB: p-CHK2
56 kDa
IB: CHK2
56 kDa
IB: α-tubulin
55 kDa
PANC-1 cells
PANC-1 cells
AD80 (μM)
Bafilomycin A1 (nM)
IB: SQSTM1/p62
62 kDa
IB: LC3B
14,19 kDa
IB: α-tubulin
55 kDa
