# Supplementary material for: The Multikinase Inhibitor AD80 Induces Mitotic Catastrophe and Autophagy in Pancreatic Cancer Cells
Source: Cancers (Basel). 2023 Jul 29;15(15):3866. doi: 10.3390/cancers15153866 (PMC10417629; doi:10.3390/cancers15153866)
Supplement: Supplementary file 1 [file cancers-15-03866-s001.zip › Figure S2.pdf]

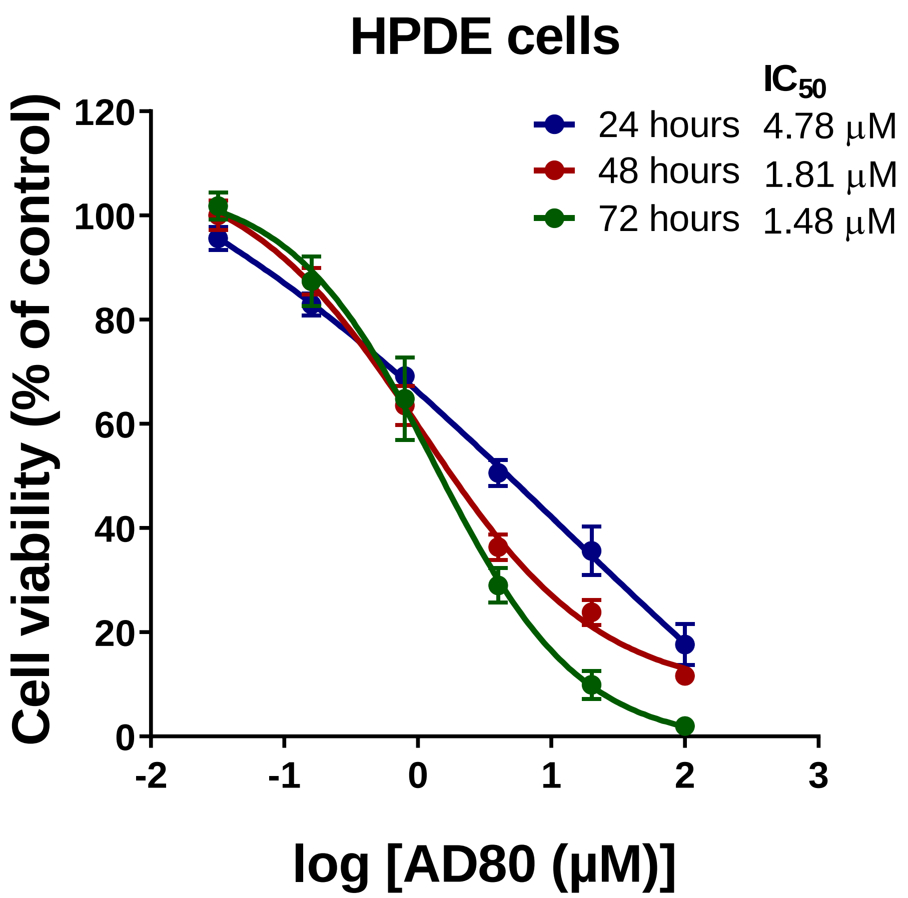

Selectivity Index (compared to HPDE cells)

Time	MIA PaCa 2 cells	PANC-1 cells	AsPC-1 cells
**24 hours**	0.39	0.16	0.11
**48 hours**	3.48	0.23	1.22
**72 hours**	18.50	0.33	4.48
