# Supplementary material for: The Multikinase Inhibitor AD80 Induces Mitotic Catastrophe and Autophagy in Pancreatic Cancer Cells
Source: Cancers (Basel). 2023 Jul 29;15(15):3866. doi: 10.3390/cancers15153866 (PMC10417629; doi:10.3390/cancers15153866)
Supplement: Supplementary file 1 [file cancers-15-03866-s001.zip › Figure S4.pdf]

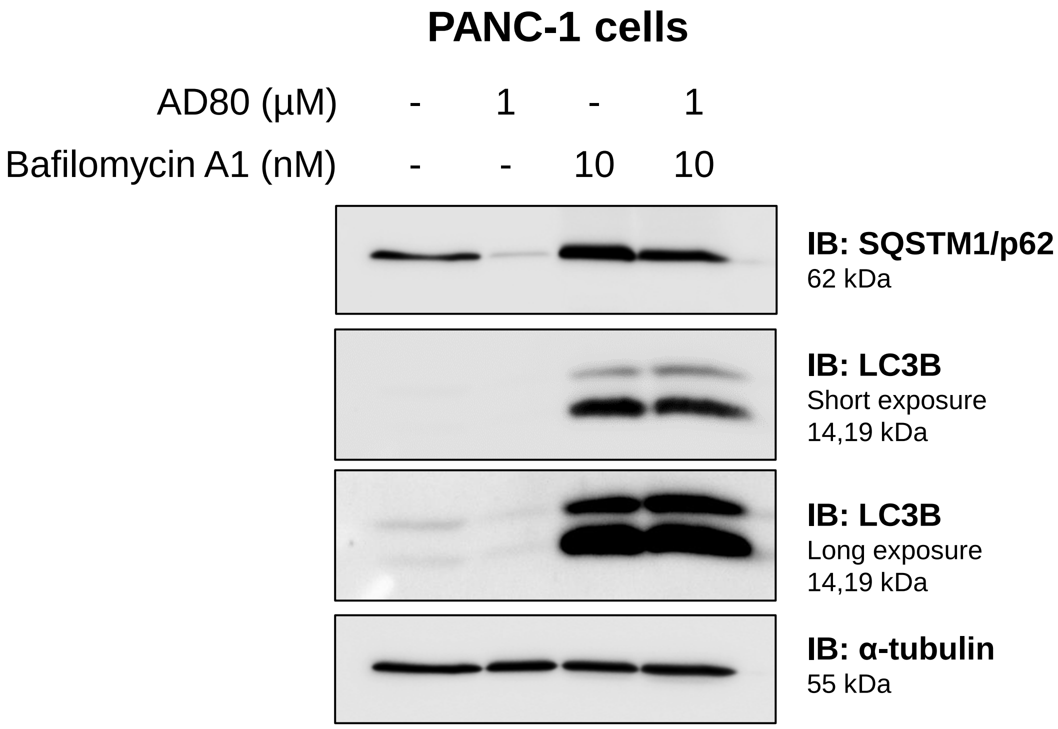
PANC-1 cells
AD80 (µM) - 1 - 1
Bafilomycin A1 (nM) - - 10 10
IB: SQSTM1/p62
62 kDa
IB: LC3B
Short exposure
14,19 kDa
IB: LC3B
Long exposure
14,19 kDa
IB: α-tubulin
55 kDa
