# Supplementary material for: The Multikinase Inhibitor AD80 Induces Mitotic Catastrophe and Autophagy in Pancreatic Cancer Cells
Source: Cancers (Basel). 2023 Jul 29;15(15):3866. doi: 10.3390/cancers15153866 (PMC10417629; doi:10.3390/cancers15153866)
Supplement: Supplementary file 1 [file cancers-15-03866-s001.zip › Supplementary Table S1.pdf]

Supplementary Table S1. Primer sequences and concentrations.

Gene	Sequence	Concentration
BBC3	FW: GACCTCAACGCACAGTACGAG<br>RV: AGGAGTCCCATGATGAGATTG	300 nM
BCL2L11	FW: ATGTCTGACTCTGACTCTCG<br>RV: CCTTGTGGCTCTGTCTGTAG	300 nM
CDKN1A	FW: TGTCACTGTCTTGTACCCTTGT<br>RV: GCCGGCGTTTGGAGTGGTAG	300 nM
CDKN1B	FW: ACTCTGAGGACACGCATTTGGT<br>RV: TCTGTTCTGTTGGCTCTTTTGTT	300 nM
GADD45A	FW: AAGGATGGATAAGGTGGGG<br>RV: CTGGATCAGGGTGAAGTGG	300 nM
PMAIP1	FW: CGCGCAAGAACGCTCAACC<br>RV: CACACTCGACTTCCAGCTCTGCT	300 nM
ACTB	FW: AGGCCAACCGCGAGAAG<br>RV: ACAGCCTGGATAGCAACGTACA	150 nM
HPRT1	FW: GAACGTCTTGCTCGAGATGTGA<br>RV: TCCAGCAGGTCAGCAAAGAAT	150 nM
