# Supplementary material for: The Multikinase Inhibitor AD80 Induces Mitotic Catastrophe and Autophagy in Pancreatic Cancer Cells
Source: Cancers (Basel). 2023 Jul 29;15(15):3866. doi: 10.3390/cancers15153866 (PMC10417629; doi:10.3390/cancers15153866)
Supplement: Supplementary file 1 [file cancers-15-03866-s001.zip › Supplementary Table S2.pdf]

Supplementary Table S2. PCR array results for autophagy-related genes upon AD80 exposure in PANC-1 cells.

Genes	AD80-treated PANC-1 cells			FC mean[1]	SD	p^2
	#1	#2	#3			
SQSTM1	0.71	0.67	0.78	0.72	0.06	0.001
HSPA8	0.64	0.85	0.76	0.75	0.11	0.016
HSP90AA1	0.93	0.73	0.82	0.83	0.10	0.038
FADD	0.79	0.95	0.75	0.83	0.10	0.048
BID	0.67	1.00	0.84	0.84	0.16	0.155
RGS19	0.94	0.86	0.75	0.85	0.10	0.056
ATG3	0.86	0.76	0.97	0.86	0.10	0.081
ATG4A	0.87	0.74	1.00	0.87	0.13	0.166
BAD	1.02	0.72	0.87	0.87	0.15	0.214
BAX	1.02	0.75	0.91	0.89	0.13	0.241
NPC1	1.15	0.70	1.04	0.97	0.23	0.813
HDAC1	1.00	0.99	0.92	0.97	0.05	0.283
SNCA	0.70	1.23	0.99	0.97	0.26	0.865
NFKB1	1.11	0.87	0.97	0.98	0.12	0.802
CASP3	0.94	1.04	0.97	0.98	0.05	0.596
ATG7	0.98	0.96	1.01	0.98	0.02	0.257
CLN3	1.28	0.85	0.85	0.99	0.25	0.957
ATG5	1.01	1.00	1.05	1.02	0.03	0.301
ATG12	0.86	1.12	1.09	1.02	0.14	0.791
RB1	1.03	1.05	1.01	1.03	0.02	0.041
PTEN	0.92	0.96	1.23	1.04	0.17	0.732
BAK1	1.19	0.79	1.17	1.05	0.22	0.700
EIF4G1	1.25	0.93	0.99	1.06	0.17	0.602
TP53	1.21	0.95	1.01	1.06	0.14	0.488
ATG9B	0.30	1.57	1.31	1.06	0.67	0.882
BCL2L1	1.28	0.98	0.98	1.08	0.18	0.476
ATG4C	1.06	0.98	1.22	1.08	0.12	0.299
PRKAA1	0.94	1.13	1.26	1.11	0.16	0.308
HDAC6	1.29	0.89	1.20	1.13	0.21	0.346
MTOR	1.03	1.06	1.31	1.13	0.16	0.214
ULK1	1.45	0.99	1.05	1.16	0.25	0.312
AKT1	1.36	1.01	1.13	1.17	0.18	0.180
CASP8	1.32	0.97	1.23	1.17	0.18	0.181
MAPK8	1.12	1.20	1.28	1.20	0.08	0.011
CTSS	1.01	1.90	0.70	1.20	0.63	0.606
BNIP3	1.05	0.98	1.59	1.21	0.33	0.341
UVRAG	1.56	0.98	1.13	1.23	0.30	0.264
DRAM2	1.21	1.14	1.34	1.23	0.10	0.018
MAP1LC3B	0.72	1.77	1.21	1.23	0.52	0.484
GABARAP	1.32	1.18	1.30	1.27	0.08	0.004
ATG16L1	1.36	1.07	1.39	1.27	0.18	0.056
ESR1	0.48	1.28	2.07	1.27	0.79	0.582
HGS	1.35	1.09	1.38	1.28	0.16	0.040
BCL2	2.42	0.61	0.81	1.28	0.99	0.653

ULK2	1.15	1.26	1.50	1.30	0.18	0.042
AMBRA1	1.39	1.19	1.33	1.30	0.10	0.007
PIK3R4	1.37	1.13	1.42	1.31	0.15	0.026
ATG9A	1.88	1.09	1.04	1.33	0.47	0.290
GABARAPL2	1.16	1.50	1.51	1.39	0.20	0.027
TGFB1	1.65	1.24	1.31	1.40	0.22	0.035
CDKN1B	1.39	1.25	1.57	1.40	0.16	0.012
ATG4B	1.34	1.34	1.57	1.42	0.13	0.006
TMEM74	1.12	1.99	1.14	1.42	0.50	0.221
HTT	1.80	1.30	1.26	1.45	0.30	0.058
BECN1	1.65	1.39	1.33	1.46	0.17	0.009
ATG4D	1.10	0.61	2.72	1.48	1.10	0.494
DRAM1	1.47	1.58	1.55	1.53	0.06	0.000
RAB24	1.57	1.36	1.75	1.56	0.20	0.008
CTSB	1.80	1.50	1.42	1.57	0.20	0.008
EIF2AK3	1.67	1.32	1.86	1.62	0.28	0.019
GABARAPL1	1.93	1.60	1.41	1.65	0.26	0.013
TGM2	1.72	1.35	2.06	1.71	0.36	0.026
ATG10	0.97	1.48	2.72	1.72	0.90	0.237
APP	2.07	1.53	1.66	1.75	0.28	0.010
RPS6KB1	1.57	1.33	2.68	1.86	0.72	0.106
LAMP1	2.42	1.54	1.78	1.91	0.46	0.026
CTSD	2.03	1.64	2.11	1.93	0.26	0.003
FAS	1.55	1.51	2.91	1.99	0.80	0.099
PIK3C3	2.04	1.60	2.52	2.05	0.46	0.017
ATG16L2	1.90	1.59	2.93	2.14	0.70	0.049
MAPK14	1.10	4.63	1.12	2.29	2.03	0.335
WIPI1	2.86	2.96	2.23	2.68	0.39	0.002
GAA	3.45	2.69	2.71	2.95	0.43	0.002
MAP1LC3A	3.52	2.84	3.29	3.22	0.34	0.000
IRGM	3.83	5.04	3.96	4.28	0.67	0.001
CXCR4	7.99	2.62	3.40	4.67	2.90	0.093
TNFSF10	9.62	3.05	5.56	6.08	3.32	0.057
CDKN2A	ND	ND	ND	-	-	-
DAPK1	ND	ND	ND	-	-	-
IFNG	ND	ND	ND	-	-	-
IGF1	ND	ND	ND	-	-	-
INS	ND	ND	ND	-	-	-
PIK3CG	ND	ND	ND	-	-	-
TNF	ND	ND	ND	-	-	-

Abbreviation: FC. Fold-change; SD. standard deviation; ND. not detected.

[1]Fold-change of vehicle-treated cells.

[2]Student *t* test.
