# Supplementary figures and images for: The Multikinase Inhibitor AD80 Induces Mitotic Catastrophe and Autophagy in Pancreatic Cancer Cells
Source: Cancers (Basel). 2023 Jul 29;15(15):3866. doi: 10.3390/cancers15153866 (PMC10417629; doi:10.3390/cancers15153866)

A

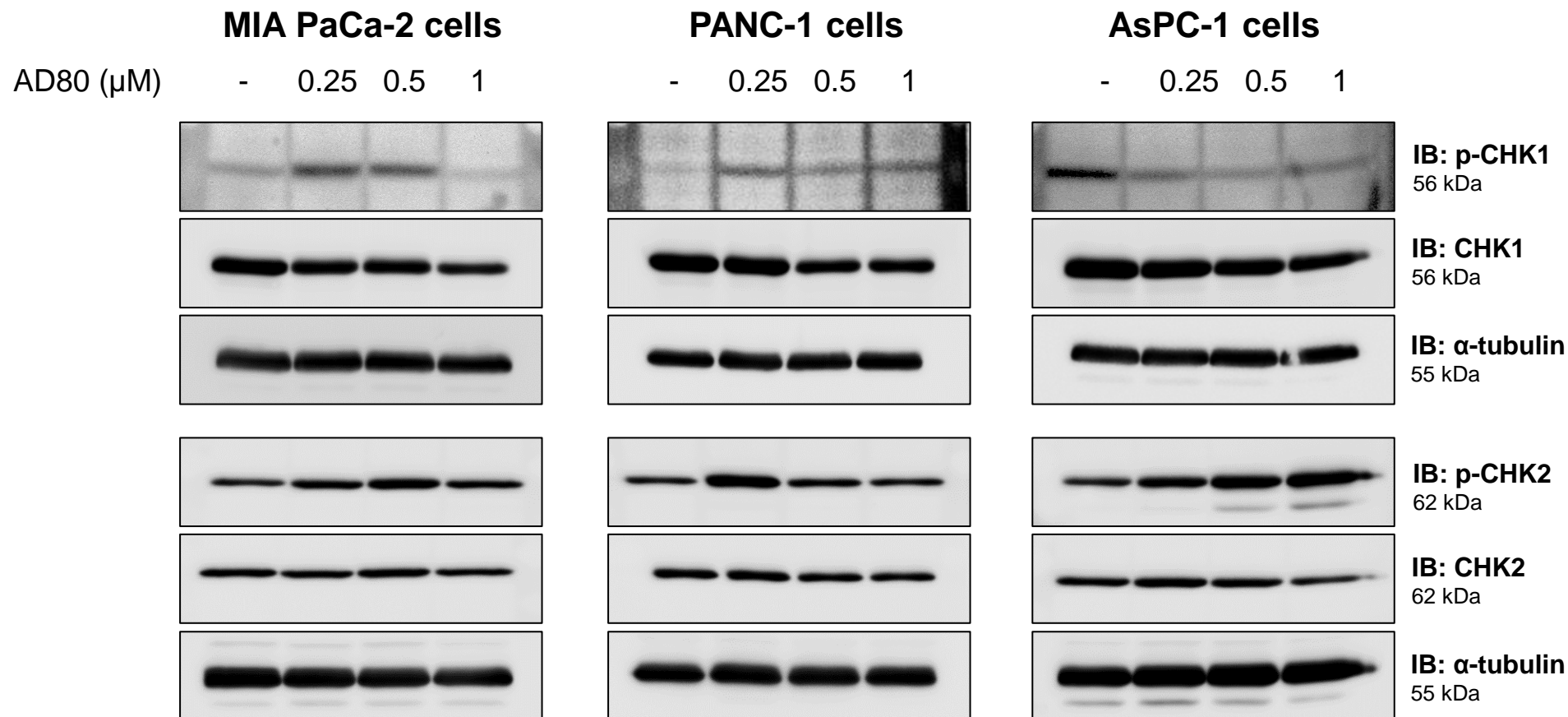

B

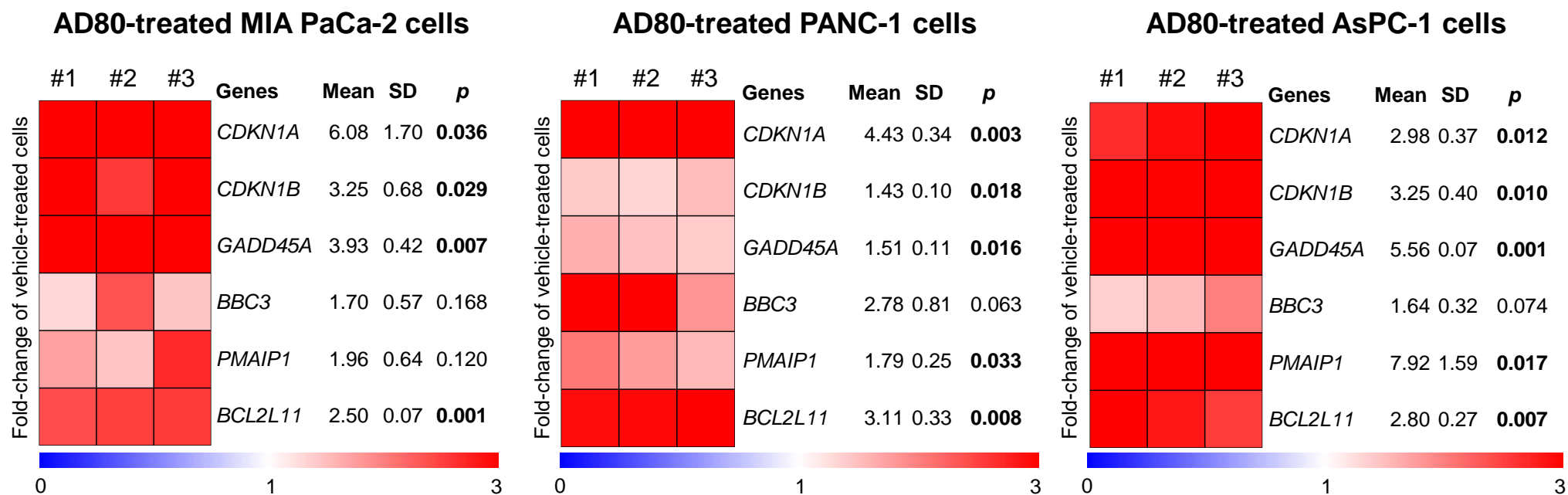

Supplement: Supplementary file 1 [file cancers-15-03866-s001.zip › Figure S3.pdf]
